# Multi-Endpoint Toxicological Assessment of Chrysin Loaded Oil-in-Water Emulsion System in Different Biological Models

**DOI:** 10.3390/nano14121001

**Published:** 2024-06-08

**Authors:** Pornsiri Pitchakarn, Pisamai Ting, Pensiri Buacheen, Jirarat Karinchai, Woorawee Inthachat, Boonrat Chantong, Uthaiwan Suttisansanee, Onanong Nuchuchua, Piya Temviriyanukul

**Affiliations:** 1 Department of Biochemistry, Faculty of Medicine, Chiang Mai University, Muang Chiang Mai, Chiang Mai 50200, Thailand; pornsiri.p@cmu.ac.th (P.P.); pensiri.bua@cmu.ac.th (P.B.); jirarat.ka@cmu.ac.th (J.K.); 2 Institute of Nutrition, Mahidol University, Salaya, Nakhon Pathom 73170, Thailand; pisamai.ting@gmail.com (P.T.); woorawee.int@mahidol.ac.th (W.I.); uthaiwan.sut@mahidol.ac.th (U.S.); 3 Department of Pre-Clinical and Applied Animal Science, Faculty of Veterinary Science, Mahidol University, Salaya, Phutthamonthon, Nakhon Pathom 73170, Thailand; boonrat.cha@mahidol.ac.th; 4 National Nanotechnology Center (NANOTEC), National Science and Technology Development Agency (NSTDA), Khlong Luang, Pathum Thani 12120, Thailand; onanong@nanotec.or.th

**Keywords:** acute toxicity, Ames test, chrysin, cytotoxicity, human health, nanoemulsion, novel food, *Drosophila* wing spot test

## Abstract

Chrysin is hypothesized to possess the ability to prevent different illnesses, such as diabetes, cancer, and neurodegenerative disorders. Nonetheless, chrysin has a low solubility under physiological conditions, resulting in limited bioavailability. In a previous study, we utilized an oil-in-water emulsion system (chrysin-ES or chrysin-NE) to encapsulate chrysin, thereby increasing its bioaccessibility and preserving its antioxidant and anti-Alzheimer’s properties. To promote the chrysin-ES as a supplementary and functional food, it was obligatory to carry out a safety assessment. Cytotoxicity testing showed that chrysin-ES was harmless, with no killing effect on 3T3-L1 (adipocytes), RAW 264.7 (macrophages), HEK293 (kidney cells), and LX-2 (hepatic stellate cells). The acute toxicity evaluation demonstrated that the 50% lethal dose (LD_50_) for chrysin-ES was greater than 2000 mg/kg BW. Genotoxicity assessments found that chrysin-ES did not induce DNA mutations in vitro or in vivo. Furthermore, chrysin and chrysin-ES exhibited anti-mutagenic properties against PhIP-induced and IQ-induced mutagenesis in the Ames test, while they inhibited urethane-, ethyl methanesulfonate-, mitomycin C-, and *N*-nitrosomethylurea-mediated mutations in *Drosophila*. The present study illustrates the safety and anti-genotoxicity properties of chrysin-ES, allowing for the further development of chrysin-based food supplements and nutraceuticals.

## 1. Introduction

Chrysin, or 5,7-dihydroxyflavone, is a dietary phytochemical classified as a flavone, which is one of the largest classes of flavonoids. It is mainly found in many plants and fruits, including *Oroxylum indicum* (L.) Kurz, *Passiflora caerulea* (blue passion flower), and *Passiflora edulis* (passion fruit), as well as propolis and honey [1,2]. Chrysin has been discovered to be a highly active flavonoid, with a wide range of pharmacological effects, such as anti-inflammation [3], anti-asthma [4], anti-cancer [5], as well as neuroprotective activities against Alzheimer’s disease (AD) and Parkinson’s disease (PD) [6]. However, the pharmaceutical efficacies of chrysin are limited by high dose intake and long-term treatment due to its low oral bioaccessibility and bioavailability. It was found that chrysin was rapidly metabolized and almost all of the chrysin uptake was poorly absorbed and eliminated in the feces (98%) [7].

Nanoemulsions, fine oil-in-water dispersions, are both kinetically and physically stable for long periods without gravitational separation, flocculation, or coalescence. Nanoemulsions are currently used to administer several medicines, including pharmaceuticals, phytopharmaceuticals, cosmeceuticals, dietary drugs, and nutraceuticals, to improve human health [8]. In addition, nanoemulsions have emerged as a powerful strategy to deliver bioactive constituents or other drugs into the human body [9]. This nanocarrier can improve the stability of the encapsulated bioactive compound from gastric degradation and is capable of enhancing membrane diffusion, which leads to sustained effects, resulting in extended release to the targeted organ, thereby improving bioaccessibility and bioavailability [8,9,10]. Our previous study developed chrysin encapsulation in an oil-in-water nanoemulsion or emulsion system (Chrysin-ES or chrysin-NE) fabricated by a heat-generated process to resolve the low bioaccessibility of chrysin. The obtained condition reduced the preparation processes and excipients and eliminated the organic solvents, resulting in a simpler preparation, increased entrapment efficiency, and improved chrysin bioaccessibility by protecting chrysin against gastric and intestinal digestion [11].

Although the most recent literature on nanoemulsions suggests that their application is safe [7], the European Food Safety Authority (EFSA) provides guidance on the risk assessment of nanomaterials to be applied in the food and feed chain, human, and animal health to ensure the safety of consumers [12]. Thus, to promote chrysin-ES in food products or nutraceutical applications, a set of toxicological studies, including in vitro and in vivo assays for toxicity and genotoxicity testing, were performed. Cytotoxicity testing is typically used to directly monitor the cellular internalization and subsequent fate of the nanoparticles. It serves as a screening tool for nanoparticle toxicity assessment before animal, clinical, and biological applications. Cytotoxicity testing on different kinds of cells that reflect the tissue body, such as the intestine, liver, kidney, adipocyte, and immune cells, was conducted [12].

Genotoxicity testing is one of the critical requirements for functional food or nutraceutical product registration. The three critical genotoxicity endpoints, including gene mutation and structural and numerical chromosome aberrations, are determined to assess the genotoxic and mutagenic potential of nanomaterials. The bacterial reverse mutation assay, or the Ames test, is the most successful and widely used to assess the genotoxicity of a substance. The capacity of the revertant bacteria to grow in the absence of the amino acid needed by the original test strain enables identification of the bacteria [13]. The histidine operon is mutated in various forms in each testing strain. Base-pair substitution mutations are present in some of the strains, such as *Salmonella Typhimurium* TA 100 and TA 1535, while frameshift mutations are present in other strains, such as *S. typhimurium* TA 98 and TA 1538, and involve the insertion or deletion of one or more bases. Each of these mutations is therefore intended to respond to mutagens that act in various ways. However, one single test is unable to detect all genotoxic endpoints. The somatic mutation and recombination test (SMART, also referred to as the wing spot test) in *Drosophila melanogaster* is one of the primary in vivo assay systems used to detect the recombinogenic effects of chemical and physical agents. It offers several methodological advantages to determine the genotoxicity of chemical substances [14,15]. The SMART in *D. melanogaster* is an in vivo system that utilizes a eukaryotic organism with metabolic equipment similar to that observed in mammalian cells [16,17]. Additionally, *D. melanogaster* can activate procarcinogens and mutagens enzymatically in vivo through CYP450-dependent activation systems [18,19].

The Ames test and the SMART assay are not only useful for genotoxicity assessment, but they also play crucial roles in measuring the anti-mutagenicity of the target compounds. Mutagenesis is the process by which a mutation in the DNA (a change in the DNA sequence or rearrangement of the chromosomes) can cause cancer and other mutation-related diseases (e.g., AD, PD, and aging) [20,21]. Various chemical environmental mutagens cause DNA damage, such as cigarette smoking, diet (fried foods, cooked meat), and cosmetics [22]. Dietary sources or synthetic compounds that can counteract mutagenic agents as potential anti-mutagens are considered a strategy in cancer chemoprevention and mutation-related diseases. The anti-mutagenic potency, coupled with antioxidant properties, is one of the mechanisms that inhibit mutagenesis [23]. Our previous study demonstrated the antioxidant ability of chrysin-ES, suggesting that it might exert anti-mutagenic activity [11].

As the potential safety of the nanoparticle materials has been considered before clinical and biological applications, this current study investigated the cytotoxicity of chrysin-ES in various normal cell lines, including human embryonic kidney cells (HEK293), human hepatic stellate cells (LX-2), mouse adipocyte fibroblasts (3T3-L1), and murine macrophage-like cells (RAW 264.7). The acute oral toxicity of chrysin-ES was also assessed in rats as an in vivo toxicity model. The genotoxicity study of the chrysin-ES was performed using a bacterial reverse mutation assay (in vitro) and the *Drosophila* wing spot test (in vivo). In addition, the potential anti-mutagenicity of chrysin-ES was investigated.

## 2. Materials and Methods

### 2.1. Chemicals and Reagents

Dulbecco’s Modified Eagle Medium (DMEM), fetal calf serum (FCS), and penicillin-streptomycin were purchased from Gibco Thermo Fisher Scientific (Grand Island, NY, USA). The fetal bovine serum (FBS) was purchased from HyClone™ (Danaher Corp., Washington, DC, USA). 2-amino-1-methyl-6-phenylimidazo (4,5-b) pyridine (PhIP), 2-Amino-3-methylimidazo(4,5-f) quinoline (IQ), and 2-(2-Furyl)-3-(5-nitro-2-furyl) acrylamide (AF-2) were purchased from FUJIFILM Wako Pure Chemical Corporation (Chern. Inc., Tokyo, Japan). 2-Aminoanthracene (2-AA), urethane, and S9 mix were purchased from Sigma-Aldrich (St. Louis, MO, USA). Chrysin, ethyl methanesulfonate (EMS), mitomycin C (MMC), sodium nitrite (NaNO_2_) and methylurea were purchased from Tokyo Chemical Industry Co., Ltd. (Tokyo, Japan).

### 2.2. Preparation of Chrysin Loaded Oil-in-Water Emulsion System (Chrysin-ES)

The chrysin loaded oil-in-water emulsion system (Chrysin-ES) was obtained from the previous study [11], as shown in the Supplementary Figure S1. Briefly, 1.74 mg of chrysin/ethanol solution was added to medium-chain triglyceride (MCT) oil, which was used as an oil phase. To form an emulsion, the oil phase (7.5% *w*/*w* MCT oil with chrysin, 7.5% *w*/*w* ethanol, and 1% *w*/*w* sorbitan monooleate) and the water phase (1% *w*/*w* polysorbate 20 (Tween-20), and 83% *w*/*w* water) were heated at 60 °C with perturbation. The two phases were mixed continuously until the emulsions were formed using an Ultra-Turrax homogenizer at a speed of 10,000 revolutions per minute, for three minutes (IKA T 25 Digital ULTRA-TURRAX^®^ Disperser, Staufen, Germany). Then, nano-sized emulsions were assorted by microfluidization at 15,000 psi for three cycles. Blank-ES was also fabricated by the same method without chrysin. 

**Figure 1:**
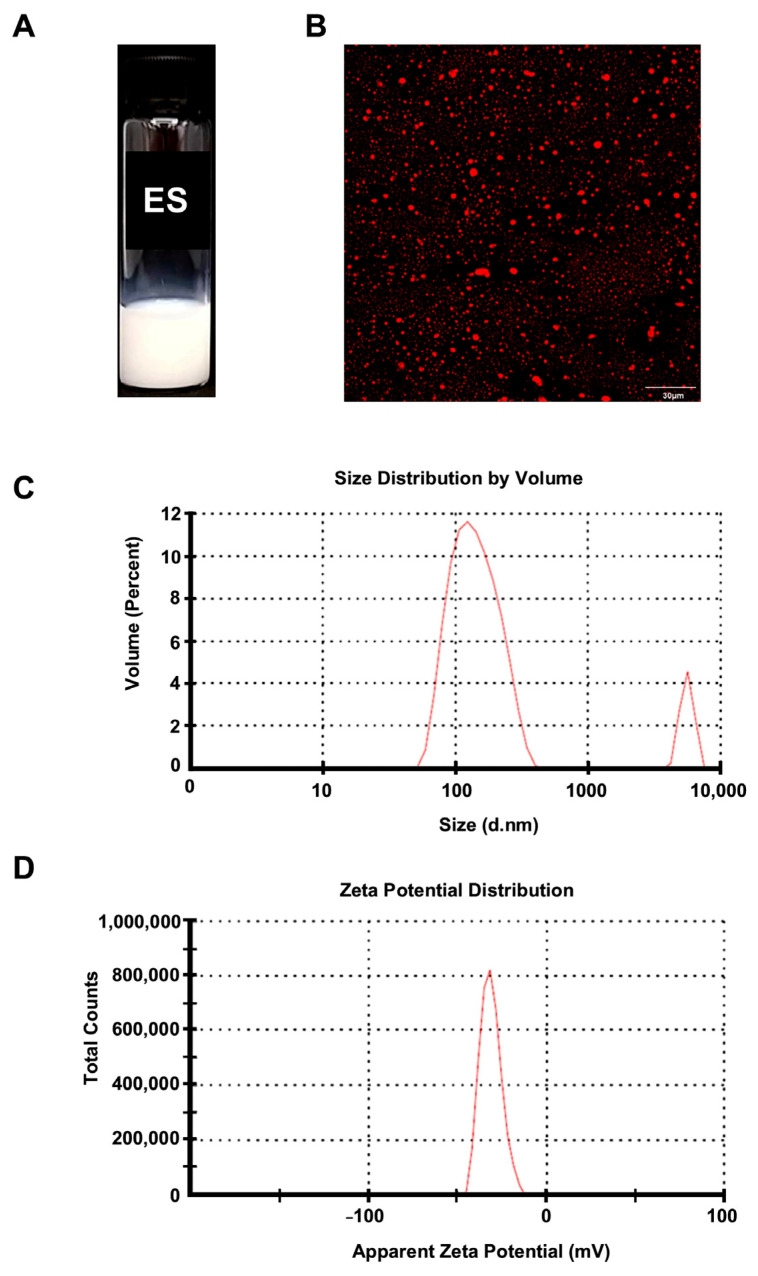
(**A**) Appearance of chrysin-ES. This chrysin-ES was obtained from the previous study (data from Ting et al., 2021 [11]). (**B**) Morphological study of chrysin-ES upon Nile red staining under confocal fluorescence microscope (scale bar = 30 µm). When using Nile red staining, chrysin-ES revealed oil droplets dispersed in an aqueous solution. The oil droplets could be the micelle formation of chrysin/MCT oil surrounded by surfactant and co-surfactant used in the formulation. (**C**) Size distribution by volume of chrysin-ES. Chrysin-ES contained heterogeneous sizes of nano-colloids about 154.4 ± 64.62 nm in diameter (88.9% volume) and microparticles about 5498 ± 626.4 nm in diameter (9.8% volume). (**D**) Zeta potential distribution of chrysin-ES used in the present study.

Quantification of chrysin content in emulsion and entrapment efficacy were performed using high-performance liquid chromatography (HPLC, Waters, Santa Clara, CA, USA) as described previously [11]. Chrysin was separated in an isocratic elution (60% *v/v* acetonitrile and 40% *v/v* water containing 1% *v/v* acetic acid) at a flow rate of 0.2 mL/min and detected at 270 nm. The entrapment efficiency (%) of chrysin in chrysin-ES was calculated as the ratio of chrysin measured in chrysin-ES divided by the initial chrysin added to emulsion and multiplied by 100 [11]. Chrysin-ES characterization, including mean particle size, particle size distribution (polydispersity index; PDI), and surface charge (zeta potential) was performed using a Zetasizer Nano ZS (Malvern Panalytical Technologies, Malvern, UK) [11]. The emulsion solution was dispersed in deionized water (DI) at a volume ratio of 1:100. All measurements were carried out in triplicate for size determination and twelve runs for zeta potential. The experiments were conducted at 25 °C, with a refractive index parameter of dispersant and material at 1.33 and 0.10, a solution viscosity at 0.8872, and a backscatter angle of 173°, as shown in Figure 1 and Supplementary Table S1). Chrysin-ES contained heterogeneous sizes of nano-colloids about 154.4 ± 64.62 nm in diameter (88.9% volume) and microparticles about 5498 ± 626.4 nm in diameter (9.8% volume). In the previous study, we referred to it as a chrysin nanoemulsion (chrysin-NE) [11]; however, in this study, we referred to it as an emulsion system (chrysin-ES), since some particles were larger than 100 nm. The microstructures of chrysin-ES were characterized by confocal fluorescence microscope (FV10i, Olympus, Center Valley, PA, USA). The oil phase was stained with Nile Red (9-diethylamino-5H-benzo[a]phenoxazine-5-one) to visualize the oil-in-water emulsion system in terms of size, surface morphology, and uniformity. The excitation and emission spectra of the Nile red dye were set at 559 nm and 670 nm, respectively (Figure 1). 

### 2.3. Cell Line and Culture Conditions

RAW 264.7 (murine macrophage-like cell), 3T3-L1 adipocyte (murine adipocyte), HEK293 (human embryonic kidney cell), and LX-2 (human hepatic stellate cell) were obtained from American Type Culture Collection (ATCC, Manassas, VA, USA). RAW 264.7 macrophages were cultured as a suspension culture in an ultra-low attachment culture dish, whereas the others were cultured as adherent cells in a cell culture flask. The cells were grown in Dulbecco’s Modified Eagle Medium (DMEM) with L-glutamine, supplemented with 10% fetal bovine serum (FBS) (RAW 264.7, HEK293, and LX-2) or 10% fetal calf serum (FCS) (3T3-L1) and 1% penicillin under 5% CO_2_, at 37 °C. When the cells reached 80% confluence, they were harvested and subjected to cytotoxicity testing.

### 2.4. Cytotoxicity Assay

RAW 264.7 (2.5 × 10^4^ cells/well), 3T3-L1 (2.5 × 10^3^ cells/well), HEK-293 (1 × 10^4^ cells/well), and LX-2 (4 × 10^3^ cells/well) were seeded in a 96-well-plate. The cells were treated with chrysin or chrysin-ES, at various doses of chrysin (2.8–90 µM), for 48 h. Blank-ES was used as a vehicle control. The amount of blank-ES was equal to chrysin-ES amount that was used for the treatment. The cytotoxicity of chrysin, chrysin-ES, and blank-ES in each cell was determined using an MTT assay. Briefly, the cells were incubated with MTT (0.5 mg/mL) reagent dissolved in serum-free medium at 37 °C for 1 h [24]. Then, the solution was gently removed, and the formazan crystal was dissolved with dimethyl sulfoxide (DMSO). The soluble formazan was measured using a microplate reader (Bio-TEK Synergy Hybrid Reader H4) at 540 nm. The cell viability is directly related to the absorbance of formazan. The percentage of cell viability was calculated using the following equation.

Cell viability (%) = (Absorbance (sample))/(Absorbance (control)) × 100

### 2.5. Acute Oral Toxicity Test in Wistar Rats

A total of 12 female Wistar rats (seven weeks old) were used for the acute toxicity study, following the OECD Guideline No. 423 [25]. Each rat was housed in an individual cage with free access to a normal diet and water and maintained in a temperature-controlled room (22 ± 3 °C), with a 12 h light/dark cycle and a relative humidity of 55 ± 10%. The animals were acclimatized to the laboratory conditions for one week before the experiments. 

After acclimatization, rats were weighed and randomly distributed into four groups, three rats per group. The mean weight of each group did not differ by more than ±20%. The starting dose was selected using the information of the test item. The oral toxicological information of the chrysin-loaded oil-in-water emulsion was not adequate, so 300 mg/kg body weight (BW) was selected for the starting dose. The dose levels of 300 and 2000 mg/kg BW were used for the study. The first and the second groups received 300 mg/kg BW (low dose) of chrysin-ES, while the third and the fourth groups received 2000 mg/kg BW (high dose) of chrysin-ES through oral gavage. All animals were monitored for any toxic indicators, such as abnormal breathing, tiredness, vomiting, muscle spasticity, seizure, hematuria, and watery diarrhea after administration, at least once during the first 30 min, periodically during the first 24 h, with special attention given during the first four hours, and daily thereafter, for a total of 14 days. The animals were general clinically observed once daily for 14 days. All animals underwent health examinations at least once a week. The examinations included changes in skin, fur/coat, eyes, and mucous membrane, the occurrence of secretions and excretions, and autonomic activity (lacrimation, piloerection, pupil size, and respiratory pattern). Changes in gait, posture, and response to handing, the presence of clonic and tonic movements, and stereotype/bizarre behavior (excessive grooming, repetitive cycling, self-mutilation, and walking backward). At the end of the experiment, all animals were sacrificed and subjected to gross necropsy.

### 2.6. In Vitro Mutagenicity and Anti-Mutagenicity Assessment Using Bacterial Reverse Mutation Assay (Ames Test)

The Ames test was performed following the OECD 471 guidelines [26]. The *S. typhimurium* strains TA 98 and TA 100 were used to detect frameshift and base-pair mutations, respectively. Each strain was tested for strain-specific markers before being used in the assay, as Maron and Ames (1983) described. The tester strains were mixed with various concentrations of chrysin, chrysin-ES, and blank-ES (5.7–22.8 µM/plate), either in the presence or absence of metabolic activation (S9 mixture, Sigma-Aldrich, St. Louis, MO, USA). Then, they were poured onto minimal glucose agar containing a trace amount of histidine, following incubation at 37 °C for 48 h. Then, the revertant colonies were counted and compared with the negative control. If the number of revertant colonies in the tested sample treatment is at least twice that of the negative control (spontaneous revertant colonies), it is considered a mutagen. Standard mutagens, including 2-aminoanthracene (2-AA) (0.5 µg/mL) and 2-(2-furyl)-3-(5-nitro-2-furyl)-acrylamide (AF-2) (0.1 µg/mL), were used as positive controls both in the presence and absence of the S9 mixture, respectively.

Furthermore, the anti-mutagenicity of the samples was performed by a similar procedure, as mentioned above. To investigate whether chrysin, chrysin-ES, and blank-ES exhibited anti-mutagenic properties, TA98 and TA100 were induced by standard mutagens, including 6-phenylimidazo [4,5-b] pyridine (PhIP) (0.1 µg/mL) and 2-Amino-3-methylimidazo [4,5-f] quinoline (IQ) (0.05 µg/mL), respectively, in the presence of the S9 mixture condition. In contrast, AF-2 (0.1 µg/mL) was used in the absence of the S9 mixture in the test of both strains. The percentage inhibition (decrease revertant colony number) was calculated as in the following equation:

Anti-mutagenicity (%) = [1 − (b/a)] × 100

a = revertant colony number induced via standard mutagen (positive control).
b = revertant colony number induced by standard mutagen in the presence of each sample treatment.

The percent of inhibition between 0–20%, 21–40%, 41–60%, and higher than 60% indicate negligible, weak, moderate, and strong anti-mutagenicity, respectively [27].

### 2.7. In Vivo Mutagenicity and Anti-Mutagenicity Assessment Using Somatic Mutation and Recombination Test in Drosophila melanogaster (Fruit Flies)

The somatic mutation and recombination test, or the wing spot test, using *D. melanogaster* was employed as an in vivo system to assess the mutagenicity and anti-mutagenicity of chrysin-ES. Two different crosses were conducted: (i) a standard cross (ST) and (ii) a high bioactivation cross (HB) [18]. The standard cross larvae were obtained from virgin female of *flr3*/*TM3*, *Ser* crossed with males of *mwh*/*mwh*, while the HB cross larvae expressing cytochrome P450 enzymes involved in xenobiotic biotransformation were obtained from virgin females of *ORR*/*ORR; flr^3^*/*In (3LR) TM3, ri p^p^sep l (3)89Aa bx^34e^ e Bd^s^* crossed with males of *mwh*/*mwh*. The flies were cultured at a constant temperature of 25 ± 1 °C and 60–70% RH at photoperiod 12:12 (light/dark). To evaluate the mutagenicity of chrysin and chrysin-ES, a hundred larva F1 progenies derived from the above crossing were fed with instant *Drosophila* medium (Formula 4-24®), Carolina, Burlington, NC, USA) containing chrysin or chrysin-ES (20–120 µg/mL). DI was used as a negative control, while 20 mM urethane (URE) was used as a positive control. The larvae were reared on each medium until pupation at 25 °C. After that, forty wings of surviving flies in each group were collected for mutant analysis. The mutant spots on the wings, including small single spots, large single spots, and twin spots, were counted. The statistical significance was calculated as previously described by Frei and Würgler [28]. 

Furthermore, the anti-mutagenicity of the samples was performed by a similar procedure as mentioned above. The larvae were fed with chrysin or chrysin-ES and mutagens (20 mM URE, 5 mM ethyl methanesulfonate (EMS), 0.05 mM mitomycin C (MMC), or *N*-nitrosomethylurea (NMU) (sodium nitrite 36 mM: methylurea 10 mM). The percentage of anti-mutagenicity of each sample was calculated as in the following equation:

Anti-mutagenicity (%) = [(a − b)/a] × 100

a = number of total spots per wing of positive mutagen control group.
b = number of total spots per wing of each experimental group.

The percent of inhibition between 0–20%, 21–40%, 41–60%, and higher than 60% indicates negligible, weak, moderate, and strong anti-mutagenicity, respectively [29]. 

### 2.8. Statistical Analysis

The data were expressed as mean ± standard deviation (SD). The experimental results were analyzed by Student’s *t*-test or one-way ANOVA with Tukey’s HSD test (as indicated in the legends) using GraphPad Prism 10.0 software. The “*p*” values less than 0.05 were considered significant. Statistical consideration for the SMART, the significances of mutant spots compared to negative or positive control were calculated following Frei and Würgler (1988), with significance levels of α = β = 0.05 [28].

## 3. Results

### 3.1. The Cytotoxicity of Chrysin, Chrysin-ES, and Blank-ES

A cytotoxicity assay is an in vitro screening test used as the first step in evaluating the ability of substances to inhibit cell viability or determine their harmfulness. This experiment determined whether chrysin-ES is safe in terms of cell viability, thus the four normal cell lines, 3T3-L1 adipocyte, RAW 264.7 macrophage, HEK-293 cells, and LX-2 cells were used to evaluate the cytotoxicity of the emulsions. According to ISO 10993-5:2009 (tests for in vitro cytotoxicity), percentages of cell viability above 80% are considered as non-cytotoxic; within 80–60% weak; 60–40% moderate; and less than 40% strong cytotoxicity [30,31]. 

The cell lines were treated with chrysin at a dose of 2.8–90 µM. As shown in Table 1, the non-toxic concentration was indicated by the inhibitory concentration at 20% of cell viability (IC_20_), while the toxic concentration was indicated by the inhibitory concentration at 50% of cell viability (IC_50_). The IC_20_ and IC_50_ of chrysin, blank-ES, and chrysin-ES were higher than 90 µM in 3T3-L1, whereas the IC_20_ of chrysin was approximately 43 ± 3.6, 53 ± 19.4, and 52 ± 13.0 µM in RAW 264.7, HEK-293 and LX-2, respectively. Noticeably, the cytotoxicity of chrysin-ES was similar to that of blank-ES in RAW 264.7 and HEK-293, with the IC_20_ of chrysin-ES at 8 ± 3.8 and 35 ± 14.1 µM in RAW 264.7 and HEK-293, respectively, and the IC_20_ of blank-ES at 9 ± 3.2 and 35 ± 12.2 RAW 264.7 and HEK-293, respectively. The IC_20_ of blank-ES and chrysin-ES was 26 ± 6.3 and 37 ± 3.2 µM in LX-2, respectively. Consistently, the IC_50_ of blank-NE and chrysin-ES in RAW 264.7, HEK-293, and LX-2 was lower than chrysin. The IC_50_ of chrysin was approximately 69 ± 3.8, >90, and >90 µM in RAW 264.7, HEK-293, and LX-2, respectively. The IC_50_ of chrysin-ES was 14 ± 3.2, 72 ± 13.1, and 61 ± 8.8 µM in RAW 264.7, HEK-293, and LX-2, respectively. While the IC_50_ of blank-ES was 27 ± 4.3, 61 ± 7.5, and 38 ± 9.9 µM in RAW 264.7, HEK-293, and LX-2, respectively. These data suggest that the oil phase component of the emulsions may influence the cytotoxicity against normal cell lines, especially RAW 264.7 and HEK-293, but not 3T3-L1.

### 3.2. Acute Toxicity of Chrysin-ES in Wistar Rat

As demonstrated in the results of cytotoxicity testing, chrysin-ES showed some toxicity, which may be due to the oil phase component. To ensure the safety of chrysin-ES, its acute oral toxicity was evaluated using Wistar rats. After being administrated with 300 and 2000 mg/kg body weight of chrysin-ES, none of the animals showed signs of toxicity, morbidity, and mortality. The health observation results for all animals showed no changes in skin and fur, eye and mucous membranes, respiratory, circulatory, autonomic and central nervous systems, somatomotor activity, and behavioral patterns. Signs of tremors, convulsions, gasping, cyanosis, vocalization, salivation, diarrhea, lethargy, sleep, and coma were not observed. For the health examination results, none of the animals exhibited changes in the skin, fur/coat, eyes, and mucous membrane, nor did they show occurrences of secretions and excretions and autonomic activity (lacrimation, piloerection, pupil size and respiratory pattern). Additionally, there were no changes in gait, posture, and response to handling, no presence of clonic and tonic movements and they did not show any stereotype/bizarre behavior (excessive grooming, repetitive cycling, self-mutilation and walking backwards) (Supplementary Table S2). 

**Figure 2:**
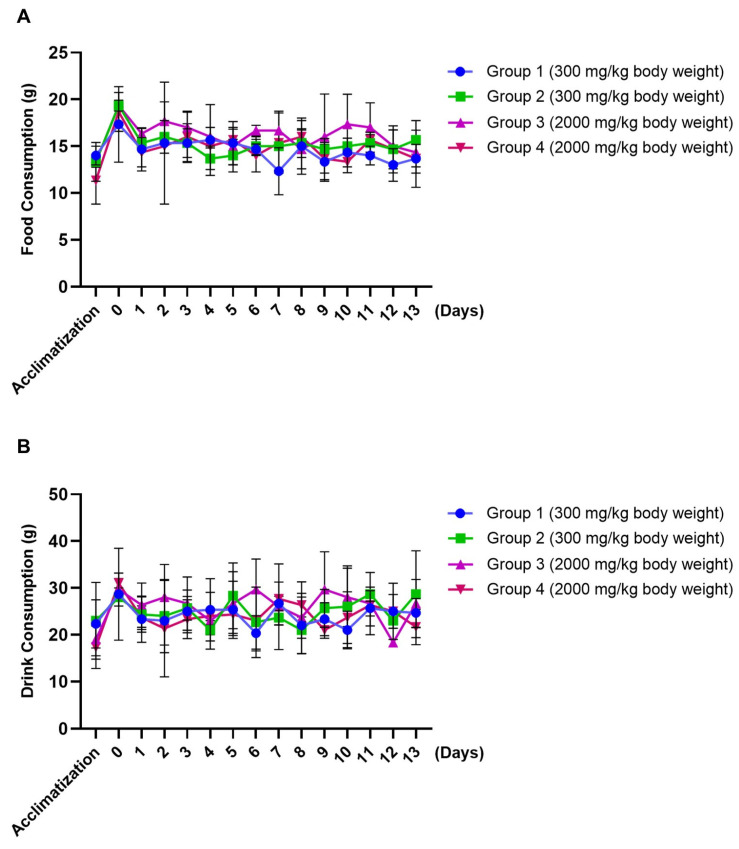
Food (**A**) and drink (**B**) consumptions of rats after administration of chrysin-ES at 300 and 2000 mg/kg BW. Values are presented as mean ± SD; N = 3.

The body weight (BW) of all animals continued to increase throughout the study (Table 2). Animal feed and drinking water consumptions were normal (Figure 2). One animal, at 300 mg/kg BW, exhibited clear fluid distension of the uterus (Supplementary Table S3). Regarding the distention of the uterus horn, it is a common finding in cycling rats. The change is a normal feature during the proestrus and estrus phases of the cycle in Wistar rat [32]. This study did not reveal abnormal gross findings that related to the test item. These results indicated that the oral LD_50_ was considered greater than 2000 mg/kg BW in the rats, suggesting the high safety of chrysin-ES. Sub-chronic and chronic toxicities studies should be further carried out to assess the long-term safety of the test item.

### 3.3. Genotoxicity Evaluation of Chrysin, Blank-ES, and Chrysin-ES in Bacteria and Fruit Flies

#### 3.3.1. In Vitro Mutagenicity of Chrysin, Blank-ES, and Chrysin-ES in *Salmonella Typhimurium*

The bacterial reverse mutation assay, or the Ames test, is a reliable bacterial assay wildly used to screen a potential mutagenic activity of suspicious compounds by measuring its ability to induce reverse mutations of selected bacteria strains [33]. *S. typhimurium* strains, which mutated in genes involved in histidine synthesis were used to investigate the potential mutagenic activity of chrysin-ES and its core materials, including pure chrysin and blank-ES. In addition, the exogenous S9 mixture was applied in the experimental condition to mimic the activation of pro-mutagens via the metabolic activation pathway. Table 3 shows the result of the negative control group. It was found that the revertant colony number of *S. typhimurium* TA98 was 26 ± 4 and 22 ± 5, and TA100 was 129 ± 12 and 125 ± 11, in the presence or absence of S9, respectively. However, the standard mutagen, 2-AA, significantly induced the revertant colonies to 459 ± 44 and 487 ± 29 in TA 98 and TA100, respectively, in the presence of S9. Similarly, AF-2 significantly increased the revertant colonies to 379 ± 30 and 439 ± 30 in TA 98 and TA100, respectively, in the absence of S9. On the other hand, the revertant colony number of *S. typhimurium* TA98 and TA100 treated with chrysin, chrysin-ES, and blank-ES (5.7–22.8 µg/plate) did not differ from the negative control either in the presence or in the absence of S9 (Table 3). The result suggests that there was no mutagenic potential of any of the samples in terms of mutagen or pro-mutagen. In line with the risk assessment of nanomaterials provided by ESFA, in vitro testing, followed by in vivo verification, has been required to approve the mutagenicity of the sample [12,24]. Regarding the chrysin forms, the chrysin-ES, and pure chrysin were further investigated for potential mutagenicity in vivo using the *Drosophila* model.

#### 3.3.2. In Vivo Mutagenicity of Chrysin and Chrysin-ES in *Drosophila melanogaster*

Chrysin and chrysin-ES were evaluated for mutagenicity using the in vivo somatic mutation and recombination test (SMART), which is a tool to detect several types of DNA damage and chromosome aberrations using *D. melanogaster*. The experiment was conducted at both normal (ST cross) and enhanced (HB cross) levels of cytochrome P450 (CYP2E1) in phase II bioactivation. The mutagenicity of chrysin and chrysin-ES was reported as the frequency of mutant spots, as shown in Table 4 and Table 5. In the ST cross, the frequency of mutant spots of the negative control group was 1.23 spots per wing, while URE significantly increased the mutant spots to 3.80 spots per wing (Table 4). Likewise, in the HB cross, the frequency of mutant spots was 0.15 and 14.50 spots per wing for the negative control and URE, respectively (Table 5). The data confirm that URE could function as both direct- and indirect-acting mutagens. The frequency of mutant spots did not differ between the chrysin or chrysin-ES treatment groups (20–120 μg/mL) and the negative control group, both in ST (Table 4) and HB cross (Table 5). These results were consistent with the Ames test, implying that chrysin and chrysin-ES were non-mutagenic in the organism, either as direct or indirect-acting mutagens.

### 3.4. Anti-Mutagenicity of Chrysin, Chrysin-ES, and Blank-ES in Bacteria and D. melanogaster

Aside from the toxicity assessment of chrysin-ES, its anti-mutagenic activity was examined. As the evidence from our previous study indicated, chrysin-ES exhibited improved antioxidant ability compared to pure chrysin [11]. It has been reported that the antioxidant activity of several compounds is correlated with their anti-mutagenic properties [34]. As a result, chrysin-ES might have potential anti-mutagenic activity to counteract the effects of mutagens. This experiment, therefore, investigated whether chrysin-ES possesses anti-mutagenic activity using the Ames test (in vitro) and SMART (in vivo) assays.

#### 3.4.1. In Vitro Anti-Mutagenicity of Chrysin, Blank-ES, and Chrysin-ES in *S. typhimurium*

To investigate the potential of the samples against mutagens, the Ames test was used as a tool to achieve the study’s objectives. In the absence of metabolic activation (−S9) (Table 6), chrysin at the used concentrations had a mild to moderate effect on inhibiting AF-2-induced mutagenesis in TA98, at approximately 35–45%. Likewise, chrysin slightly inhibited AF-2-induced mutagenesis by 32–37% in TA100 (5.7–22.8 µM/plate). Interestingly, chrysin-ES effectively inhibited AF-2-induced TA98 by 28–78% in a dose-dependent manner (5.7–22.8 µM/plate) but had a negligible effect on TA 100 (Table 6). Remarkably, chrysin was highly effective against mutagens under the metabolic activation (+S9) condition, as shown in Table 7. Chrysin strongly inhibited PhIP-induced TA98 and IQ-induced TA100 by 81–89% and 78–94%, respectively, at concentrations ranging from 1.4 to 5.7 µM/plate. Similarly, chrysin-ES had a strong mutagenic inhibition against PhIP-induced TA98 and IQ-induced TA100 by 66–89% and 89–97%, respectively, at concentrations ranging from 1.4–5.7 µM/plate (Table 7). The results indicated that chrysin and chrysin-ES exhibited anti-mutagenic activity, particularly on mutagens requiring metabolic activation (indirect-acting mutagens). However, the tester strains were limited to mutation type, representing only point mutations (frameshift and base substitutions). Thus, the potential of samples against mutagens acting via different mechanisms was further investigated in the *Drosophila* model.

#### 3.4.2. In Vivo Anti-Mutagenicity of Chrysin and Chrysin-ES in *Drosophila melanogaster*

SMART, using *Drosophila,* can detect a wide range of DNA mutations, as well as chromosome breaks and chromosomal aberrations. Various standard mutagens, including urethane (URE), ethyl methanesulfonate (EMS), mitomycin C, and *N*-nitrosomethylurea (NMU, a product from a combination of sodium nitrite methylurea), were used to investigate the anti-mutagenicity effects of chrysin and chrysin-ES. Chrysin and chrysin-ES (20–120 µg/mL) significantly inhibited URE-induced mutant spots of adult wings by 86–94% and 83–95%, respectively, in the ST cross, as shown in Table 8. Similar effects were observed in the HB cross (Table 9). Chrysin and chrysin-ES (20–120 µg/mL) significantly decreased the mutant spots induced by URE, at approximately 84–92% and 79–94%, respectively, indicating that chrysin and chrysin-ES had strong anti-mutagenic effects against URE-induced mutagenesis in *Drosophila,* both in the ST cross and the HB cross.

The anti-mutagenic effects of chrysin and chrysin-ES against MMC-mediated mutations are shown in Table 10 and Table 11. In the ST cross, the data demonstrated that the mutagenicity of MMC could be inhibited by chrysin at 40–80 µg/mL with a weak level of inhibition, at approximately 30% inhibition, and at 120 µg/mL with a strong level of inhibition, at approximately 77% inhibition, while with the lowest dose (20 µg/mL) of chrysin, the inhibition effect on MMC-induced mutagenesis was not observed (Table 10). The lowest dose of chrysin-ES (20 µg/mL) could be observed to inhibit mutagenicity against MMC by 39% inhibition. In addition, 40 µg/mL and 80 µg/mL of chrysin-ES decreased the MMC mutagenicity, with moderate levels of inhibition (50% and 53%, respectively), while the MMC mutagenicity was strongly inhibited by 120 µg/mL chrysin-ES (83% inhibition) (Table 10). In the HB cross, chrysin at 40, 80, and 120 µg/mL exhibited a strong inhibitory effect against MMC at 60%, 79%, and 88% inhibition, respectively (Table 11), while 20 µg/mL of chrysin had a negligible effect against MMC (5% inhibition). Moreover, all concentrations of chrysin-ES showed a strong inhibitory effect against MMC, within the range of 92–97% inhibition (Table 11). These findings indicate that chrysin and chrysin-NE could decrease the mutagenicity of MMC, and their activity was highly effective in the HB cross. Moreover, chrysin-ES had an inhibitory effect against MMC, greater than that of chrysin in the ST and HB crosses.

The anti-mutagenic effect of chrysin and chrysin on EMS-induced mutagenesis is shown in Table 12 and Table 13. In the ST cross, the data revealed that low doses of chrysin (20 µg/mL) could not inhibit the mutagenicity of EMS (19% inhibition), but chrysin at concentrations of 40–120 µg/mL showed a moderate inhibition of EMS mutagenicity, within the range of 42–49% (Table 12). Whereas chrysin-ES had a weak inhibition against EMS, inhibitions of 26% and 39% were observed at concentrations of 20 and 40 µg/mL, respectively. Additionally, chrysin-ES decreased EMS mutagenicity, with moderate inhibitions of 42% and 55% at concentrations of 80 and 120 µg/mL, respectively (Table 12). In the HB cross (Table 13), chrysin at concentrations of 20 and 40 µg/mL could decrease the EMS-induced mutant spots, with inhibition ranging between 25% and 31% (weak inhibition), respectively, while a strong effect of chrysin was observed at concentrations of 80 and 120 µg/mL, with inhibition ranging between 60% and 76%, respectively. Chrysin-ES showed weak (40% inhibition) and moderate (51% inhibition) effects against the mutagenicity of EMS, at concentrations of 20 and 40 µg/mL, respectively. A strong effect on inhibiting EMS-induced mutagenesis, with approximately 51% and 76% inhibition, was observed at concentrations of 80 and 120 µg/mL, respectively (Table 13). These findings indicate that high doses of chrysin and chrysin-NE effectively decrease the mutagenicity of EMS.

The modulating effect of chrysin and chrysin-ES on the *N*-nitrosomethylurea (NMU) formation is shown in Table 14 and Table 15. In the ST cross, all concentrations of chrysin (20–120 μg/mL) and chrysin-NE (20–120 μg/mL) strongly inhibited the mutagenicity caused by NMU formation within the range of 89–91% inhibition and 85–95% inhibition, respectively (Table 14). In the HB cross, chrysin at concentrations of 40–120 μg/mL had a strong effect against NMU formation-induced mutagenesis, within the range of 80–95% inhibition, while a concentration of 20 μg/mL of chrysin showed a moderate inhibitory effect (Table 15). Chrysin-ES at concentrations of 20–120 μg/mL exhibited a moderate to strong inhibitory effect within the range of 48–84% inhibition. These results indicate that chrysin and chrysin-NE could decrease the mutagenicity caused by NMU formation. Taken together the results suggested that chrysin and chrysin-ES exerted anti-genotoxicity ability. The efficacy of chrysin against mutagens may vary depending on the form of chrysin, the class of mutations, and their mechanism of action.

## 4. Discussion

Chrysin, a naturally occurring flavonoid, has various health-promoting properties, such as antioxidant, anti-inflammatory, anti-diabetic, and anti-cancer activities [2]. Chrysin exerts anti-cancer properties through the induction of apoptosis and inhibition of cancer cell migration in various types of cancer [35,36,37,38,39,40]. Moreover, the compound shows neuroprotective properties by improving neurogenesis in aging-mediated memory loss induced by oxidative stress and inflammation [40]. It has been reported that chrysin has anti-hyperglycemic and anti-diabetic effects in diabetic mice [41]. It also plays a vital role in neurological disorders such as AD and PD. However, the health-promoting and alleviating benefits of chrysin are restricted by its poor bioavailability, which includes low absorption and rapid rates of metabolism and excretion [42]. To improve its bioaccessibility, chrysin loaded with an oil-in-water emulsion system was developed. The various features of emulsion, including kinetic stability, high surface-active area, and stabilizing properties, can improve the dispersibility and bioavailability of active compounds for nutrient delivery systems [8,9,43]. For these reasons, oil-in-water emulsions are applicable for the oral delivery of lipophilic nutraceuticals. Our previous study demonstrated that the encapsulation of chrysin using an oil-in-water emulsion system (chrysin-ES or chrysin-NE), fabricated by a heat-generated process increased the entrapment efficiency and enhanced the bioaccessibility of chrysin, which was more tolerant of gastrointestinal digestion, being significantly absorbed by human intestinal cells compared to pure chrysin [11]. Moreover, the antioxidant and anti-AD properties of chrysin-ES were retained. The finding suggests the possibility of developing chrysin-loaded oil-in-water emulsions for application in the food and nutraceutical industries. 

Nanoemulsions or emulsion systems are composed of natural and/or synthetic materials such as oils, surfactants, and cosurfactants. However, some core materials, such as synthetic surfactants (e.g., Tweens and Spans) may promote irritation or toxicity at high levels of use [44]. Therefore, the potential adverse effects of nanomaterials are a significant concern for their use in both animals and humans. Regarding the further usage of chrysin-ES in food or nutraceutical applications, the present study evaluated its safety both in vitro and in vivo by cytotoxicity using cell cultures and acute toxicity using rat models, respectively. Moreover, the genotoxicity of chrysin-ES in bacteria and *Drosophila* was assessed. The cytotoxicity of chrysin-ES and its core materials (chrysin and blank-ES) was investigated in the targeted cells following the EFSA guidelines, including RAW 264.7 (murine macrophage-like cell), 3T3-L1 adipocyte (murine adipocyte), HEK293 (human embryonic kidney cell), and LX-2 (human hepatic stellate cell), which represented the targeted organs in the human body. It was found that the cytotoxicity of chrysin was increased following the emulsion formation, implying that the oil phase component might affect cell survival, particularly in RAW264.7. Thus, the toxicity of the oil phase component should be considered. Interestingly, when chrysin-ES (2000 mg/kg BW) was orally administered to Wistar rats, the survival and general clinical observations indicated that chrysin-ES did not show acute toxicity in the rats. From these data, the estimated LD50 of chrysin-ES might be greater than 2000 mg/kg BW. According to Yao et al. [45], 40% mortality was observed in Sprague Dawley rats after a single oral chrysin administration (5000 mg/kg BW), thereby the LD50 was approximately at 5000 mg/kg BW. Although we did not increase chrysin-ES to an extremely high dose, at 5000 mg/kg BW, in terms of novel foods, the encapsulation of chrysin using an oil-in-water emulsion was found to be safe in rats, suggesting that it might be safe to use in other animals and humans. 

Although previous studies have reported the non-mutagenicity of chrysin and MCT, which are the main components of the nanoemulsion [46,47,48], the potential genetic damage of chrysin-ES has not been assessed. Thus, we investigated the possible genotoxicity of chrysin-ES. Genotoxicity is the ability of harmful agents to damage the genetic information in cells, causing mutations that eventually lead to cancer and non-cancerous diseases, such as neurodegenerative and cardiovascular diseases [20]. The products that are purposed for consumption should be evaluated for mutagenicity potential. The bacterial reverse mutation, or the Ames test, using the *S. typhimurium* strains TA98 and TA100, which can be used to detect frameshift mutations and base-pair substitutions, respectively, was performed to screen the mutagenicity of chrysin-ES. Some agents are pro-mutagens, which require biotransformation or metabolic activation via cytochrome-P450 to become more genotoxic products (active mutagens). Thus, the S9 mixture was applied as an exogenous metabolic activation in the test system due to the lack of a metabolic activation system in bacteria. The S9 mixture contains microsomal enzymes, including cytochrome P450 isoform (phase I metabolism), and cytosolic enzymes containing a major part of the activities of transferases (phase II metabolism) [49,50]. This study reveals that chrysin-ES, as well as its core materials, including chrysin and blank-ES, did not induce mutations in the *S. typhimurium* strains TA98 and TA100 in the presence and absence of metabolic activation, implying that chrysin-ES was neither a pro-mutagen nor a mutagen. In addition, we further evaluated the genotoxicity of chrysin-ES using the wing spot test in *Drosophila*, which is the in vivo high-throughput genotoxicity test. The test shows more advantages by detecting several types of mutations, including base-pair substitutions, frameshift mutation, DNA breaks, DNA crosslinks, and chromosomal aberrations [15,16]. The mutagenicity of chrysin-ES was performed both in the ST cross and the HB cross in *Drosophila*. The HB cross in the flies has been developed for high metabolic capacity, expressing a high constitutive level of cytochrome P450 to activate pro-mutagens, similar to mammals [18,19]. The potential mutagenicity of chrysin-ES was not observed either in the ST cross or the HB cross, which was consistent with the result from the Ames test. Taken together, the toxicity evaluation of chrysin-ES indicated that chrysin-ES was non-toxic and non-mutagenic, suggesting chrysin-ES might be safe for applications in animals and humans.

As evidenced by our previous study, chrysin-ES improved antioxidant ability compared to standard chrysin [11]. To our knowledge, there is a correlation between antioxidant activity and anti-mutagenic properties [34]. Therefore, chrysin-ES might possess potential anti-mutagenic activity to counteract or ameliorate the activity of mutagens. Therefore, the anti-mutagenesis of chrysin-ES against various standard mutagens found in daily life was performed. Furylfuramide (AF-2), a synthetic nitrofuran derivative, was used as a food preservative but was forbidden because of its carcinogenicity. 2-Amino-1-methyl-6-phenylimidazo [4,5-b]pyridine (PhIP) and 2-Amino-3-methylimidazo [4,5-f]quinoline (IQ) are the predominant heterocyclic amines found in cooked meat and classified as a possible mutagen (Group 2A) and a probable mutagen (Group 2B) in humans, respectively [51,52]. In the Ames test, AF-2 was used as a mutagen in the absence of metabolic activation in the *S. typhimurium* strains TA98 and TA100. Chrysin mildly inhibited AF-2-mediated mutations in both TA98 and TA100, while chrysin-ES had a mild to strong ability to inhibit AF-2-induced mutations only in TA98, in a dose-dependent manner. Similarly, blank-ES also exhibited mild to strong anti-mutagenicity in TA 98, and its activity was not observed in TA 100. Noticeably, the activity of chrysin was diminished in the form of emulsion, suggesting that the components of emulsions, especially MCT oil, might influence the activity of chrysin-ES against AF-2-induced mutagenesis. For the metabolic-activated mutagens, chrysin and chrysin-ES exhibited strong anti-mutagenic activity against PhIP-induced TA98 and IQ-induced TA100, even at the low concentrations, whereas blank-ES had strong activity at above 5.70 µM/plate. The data suggest that chrysin and chrysin-ES seemed to exert anti-mutagenicity against pro-mutagens (PhIP and IQ) rather than direct mutagens (AF-2). As the SMART assay is extensively capable of assessing different types of mutations, we further investigated the anti-mutagenic activity of chrysin-ES against the well-established mutagens or carcinogens, including urethane (URE), ethyl methanesulfonate (EMS), mitomycin C (MMC), and *N*-nitrosomethylurea (NMU), using the *Drosophila* model. Accordingly, anti-mutagenesis is one of the mechanisms to prevent cancer by inhibiting mutation through various mechanisms such as the inhibition of mutagen uptake, deactivation and detoxification of the mutagen, the blocking of mutagen–DNA binding, and improved DNA repair [53,54]. URE, a Group 2B mutagen, is metabolized by CYP2E1 to vinyl carbamate, which can cause DNA damage, leading to deletion and point mutations [55]. Our study demonstrated the strong anti-mutagenic activity of chrysin and chrysin-ES both in the normal and high expression of the CYP450 conditions. Accordingly, previous studies reported that chrysin was a CYP2E1 inhibitor [56,57]. The data could imply that chrysin and chrysin-ES could inhibit URE-induced mutagenesis via CYP2E1 inhibition. MMC is widely used as a chemotherapy agent and an antibacterial spectrum. MMC causes interstrand and intrastrand DNA crosslinks, leading to chromosome recombination [58]. In addition, MMC induces hydroxy free radicals through metabolic activation, via the NADPH-cytochrome P-450 reductase system, which damages DNA. The mutagenicity of MMC was inhibited by chrysin and chrysin-ES, especially in the high level of metabolic activation system. The data could imply that chrysin and chrysin-ES may inhibit MMC-induced mutagenesis by acting as blocking agents and antioxidants. EMS is an alkylating agent that can directly react with the base of DNA by guanine alkylation, resulting in G:C to A:T transitions, eventually causing point mutation [58]. Chrysin and chrysin-ES also exerted an anti-mutagenic activity in EMS-induced mutagenesis, especially in the high metabolic activation condition. Based on the mechanism of action of EMS, chrysin and chrysin-ES probably block the binding of the mutagen to DNA, decreasing the formation of DNA adducts. *N*-nitroso compounds (NOC), alkylating agents, are potent carcinogens that do not require metabolic activation [59]. NOC can be found in nitrite-preserved food, especially processed meat. We investigated the anti-mutagenicity of chrysin and chrysin-ES against NMU, which is produced by sodium nitrite and methylurea via the nitrosation in vivo. Both chrysin and chrysin-ES could inhibit the endogenous formation of NMU, as shown by the strong anti-mutagenic activity both in the normal and high bioactivation cross systems, implying that inhibiting the formation of nitroso compounds may be one of the possible mechanisms by which chrysin and chrysin-ES prevent mutagenesis.

## 5. Conclusions

Together, the data show that the chrysin-loaded oil-in-water emulsion system developed by our procedure may be safe for further application in animals and humans, as evidenced by non- or sub-toxicity to the normal cell line, no adverse effects in rats (acute toxicity testing), and non-genotoxicity, evaluated in bacteria and *Drosophila* models. This study provides scientific evidence that would be useful for further safety studies in animal models and clinical trials to ensure the safety of the emulsion system. Furthermore, we highlighted the high potential anti-mutagenic activity of chrysin-ES, which might support the development of chrysin-ES as a novel ingredient for the prevention of cancer or other diseases derived from DNA mutations.

## Figures and Tables

**Table 1:** Cytotoxicity of chrysin, chrysin-ES, and blank-ES against various types of normal cell lines (3T3-L1, RAW 264.7, HEK-293, and LX-2).

Sample	IC_20_ (µM)				IC_50_ (µM)			
	3T3-L1	RAW 264.7	HEK-293	LX-2	3T3-L1	RAW 264.7	HEK-293	LX-2
Chrysin	>90	43 ± 3.6	53 ± 19.4	52 ± 13.0	>90	69 ± 3.8	>90	>90
Chrysin-ES	>90	8 ± 3.8 ^c^	35 ± 14.1 ^a^	37 ± 3.2	>90	14 ± 3.2 ^c^	72 ± 13.1	61 ± 8.8 ^c^
Blank-ES	>90	9 ± 3.2 ^c^	35 ± 12.2 ^b^	26 ± 6.3	>90	27 ± 4.3 ^c,d^	61 ± 7.5 ^b^	38 ± 9.9 ^c,e^

IC_20_ and IC_50_ are the concentrations required to decrease cell viability by 20% and 50%, respectively. The values (µM) are expressed as mean ± SD of three independent experiments. ^a^ *p* < 0.05, ^b^ *p* < 0.01, ^c^ *p* < 0.001 compared to chrysin, ^d^ *p* < 0.05, ^e^ *p* < 0.01 compared to blank-ES.

**Table 2:** Effects of chrysin-ES on body weight of rats in acute toxicity study on days 1, 7, and 14.

Group	Chrysin-ES (mg/kg BW)	Body Weight (g)					Body Weight Change (%)
		Acclimatization	Day 0	Day 7	Day 14	Terminate	
1	300	199 ± 5.0	200 ± 7.6	222 ± 15.3	239 ± 17.4	235 ± 15.5	19.38 ± 4.6
2	300	196 ± 2.5	200 ± 2.5	221 ± 1.0	240 ± 8.1	236 ± 7.8	19.62 ± 2.9
3	2000	191 ± 3.1	203 ± 1.5	234 ± 6.2	248 ± 7.0	244 ± 6.2	22.22 ± 3.8
4	2000	192 ± 2.0	202 ± 3.5	225 ± 7.5	237 ± 2.5	232 ± 2.8	17.38 ± 2.1

Values were presented as mean ± SD; N = 3.

**Table 3:** The mutagenicity of chrysin, blank-ES, and chrysin-ES in the presence and absence of metabolic activation (S9 mix) using *S. typhimurium* strains TA 98 and TA 100.

Treatments	Number of Revertant Colonies ^a^			
	TA98		TA100	
	+S9	−S9	+S9	−S9
**DMSO (negative control)**	26 ± 4	22 ± 5	129 ± 12	125 ± 11
**2-AA (0.5 µg/plate)**	459 ± 44	ND	487 ± 29	ND
**AF-2 (0.1 µg/plate)**	ND	379 ± 30	ND	439 ± 30
**Chrysin**				
5.7 µM/plate	27 ± 7	22 ± 6	136 ± 12	120 ± 6
11.4 µM/plate	26 ± 8	23 ± 5	131 ± 7	115 ± 7
22.8 µM/plate	27 ± 8	23 ± 7	136 ± 8	117 ± 4
**Blank-ES**				
5.7 µM/plate	26 ± 9	22 ± 5	113 ± 10	119 ± 7
11.4 µM/plate	26 ± 8	21 ± 3	136 ± 6	124 ± 8
22.8 µM/plate	31 ± 8	26 ± 3	132 ± 10	128 ± 6
**Chrysin-ES**				
5.7 µM/plate	26 ± 8	24 ± 7	128 ± 8	121 ± 9
11.4 µM/plate	25 ± 6	23 ± 6	131 ± 10	117 ± 4
22.8 µM/plate	30 ± 9	29 ± 7	131 ± 8	130 ± 10

^a^ The data are indicated as mean ± SD of revertant colonies of six plates per treatment, *p* < 0.05. ND: not determined.

**Table 4:** The mutagenicity of chrysin and chrysin-ES reported as wing spot induction on *D. melanogaster* derived from trans-heterozygous *mwh*+/+*flr^3^* larvae of the ST cross.

Samples	Frequency of Mutant Spots per Individual (Number of Spots) ^a^			
	Small Single (m = 2)	Large Single (m = 5)	Twin (m = 5)	Total Spots (m = 2)
**DI (negative control)**	1.23 (49)	0 (0)	0 (0)	1.23 (49)
**URE (20 mM)**	3.73 (149) +	0.08 (3) +	0 (0) i	3.80 (152) +
**10% EtOH in PBS**	1.00 (40) −	0 (0) i	0 (0) i	1.00 (40) −
**Chrysin**				
20 µg/mL	0.38 (15) −	0 (0) i	0 (0) i	0.38 (15) −
40 µg/mL	0.35 (14) −	0.03 (1) i	0 (0) i	0.38 (15) −
80 µg/mL	0.58 (23) −	0 (0) i	0 (0) i	0.58 (23) −
120 µg/mL	0.83 (33) −	0.08 (3) i	0 (0) i	0.90 (36) −
**Chrysin-ES**				
20 µg/mL	0.43 (17) −	0 (0) i	0 (0) i	0.43 (17) −
40 µg/mL	0.45 (18) −	0.08 (3) i	0 (0) i	0.53 (21) −
80 µg/mL	0.60 (24) −	0 (0) i	0 (0) i	0.60 (24) −
120 µg/mL	1.28 (51) −	0.08 (3) i	0 (0) i	1.35 (54) −

^a^ Statistical diagnoses using estimation of spot frequencies and confidence limits according to Frei and Würgler (1988) [28] for comparison with deionized water (DI); “+” = positive; “−” = negative; “i” = inconclusive. Probability levels: α = β = 0.05. One-sided statistical test. URE = urethane; EtOH = ethanol; PBS = phosphate buffer saline, pH 7.4.

**Table 5:** The mutagenicity of chrysin and chrysin-ES reported as wing spot induction on *D. melanogaster* derived from trans-heterozygous *mwh*+/+*flr^3^* larvae of the HB cross.

Samples	Frequency of Mutant Spots per Individual (Number of Spots) ^a^			
	Small Single (m = 2)	Large Single (m = 5)	Twin (m = 5)	Total Spots (m = 2)
**DI (negative control)**	0.15 (6)	0 (0)	0 (0)	0.15 (6)
**URE (20 mM)**	10.10 (202) +	3.10 (62) +	1.30 (26) +	14.50 (290) +
**10% EtOH in PBS**	0.08 (3) −	0 (0) i	0 (0) i	0.08 (3) −
**Chrysin**				
20 µg/mL	0.18 (7) i	0.03 (1) i	0.03 (1) i	0.23 (9) i
40 µg/mL	0.13 (5) i	0.03 (1) i	0 (0) i	0.15 (6) i
80 µg/mL	0.13 (5) i	0.03 (1) i	0 (0) i	0.15 (6) i
120 µg/mL	0.08 (3) −	0 (0) i	0 (0) i	0.08 (3) −
**Chrysin-ES**				
20 µg/mL	0.18 (7) i	0 (0) i	0 (0) i	0.18 (7) i
40 µg/mL	0.18 (7) i	0 (0) i	0 (0) i	0.18 (7) i
80 µg/mL	0.30 (12) i	0 (0) i	0 (0) i	0.30 (12) i
120 µg/mL	0.05 (2) −	0 (0) i	0 (0) i	0.05 (2) −

^a^ Statistical diagnoses using estimation of spot frequencies and confidence limits according to Frei and Würgler (1988) [28] for comparison with deionized water (DI); “+” = positive; “−” = negative; “i” = inconclusive. Probability levels: α = β = 0.05. One-sided statistical test. URE = urethane; EtOH = ethanol; PBS = phosphate buffer saline, pH 7.4.

**Table 6:** The anti-mutagenicity of chrysin, blank-ES, and chrysin-ES on the standard mutagen treated in the absence of metabolic activation (−S9), using *S. typhimurium* TA98 and TA100.

Sample	TA98		TA100	
	AF-2 (0.1 µg/Plate)		AF-2 (0.1 µg/Plate)	
	Number of Revertant Colonies ^a^	Inhibitory Classification	Number of Revertant Colonies ^a^	Inhibitory Classification
**Standard mutagen**	252 ± 12	ND	396 ± 11	ND
**Blank-ES**				
5.7 µM/plate	193 ± 24 (23) ^b^	Mild	349 ± 40 (6)	Negligible
11.4 µM/plate	120 ± 15 (52)	Moderate	400 ± 13 (−8)	NA
22.8 µM/plate	36 ± 8 (86)	Strong	407 ± 44 (−10)	NA
**Chrysin**				
5.7 µM/plate	166 ± 23 (34)	Mild	252 ± 14 (32)	Mild
11.4 µM/plate	140 ± 39 (45)	Moderate	250 ± 6 (32)	Mild
22.8 µM/plate	164 ± 56 (35)	Mild	235 ± 20 (37)	Mild
**Chrysin-ES**				
5.7 µM/plate	182 ± 23 (28)	Mild	321 ± 33 (13)	Negligible
11.4 µM/plate	141 ± 26 (44)	Moderate	338 ± 26 (9)	Negligible
22.8 µM/plate	54 ± 18 (78)	Strong	375 ± 36 (−1)	Negligible

^a^ The data are indicated as mean ± SD of the number of revertant colonies of triplicate plates per treatment from two independent experiments. ^b^ Values in brackets indicate % mutagenic inhibition. ND: not determined. NA: not applicable.

**Table 7:** The anti-mutagenicity of chrysin, blank-ES, and chrysin-ES on the standard mutagen treated in the presence of metabolic activation (+S9), using *S. typhimurium* TA98 and TA100.

Sample	TA98		TA100	
	PhIP (0.1 µg/Plate)		IQ (0.05 µg/Plate)	
	Number of Revertant Colonies ^a^	Inhibitory Classification	Number of Revertant Colonies ^a^	Inhibitory Classification
**Standard mutagen**	400 ± 11	ND	730 ± 39	ND
**Blank-ES**				
5.7 µM/plate	169 ± 45 (53) ^b^	Moderate	497 ± 36 (32)	Mild
11.4 µM/plate	104 ± 33 (71)	Strong	335 ± 19 (54)	Strong
22.8 µM/plate	81 ± 16 (78)	Strong	198 ± 63 (73)	Strong
**Chrysin**				
0.35 µM/plate	214 ± 9 (46)	Moderate	328 ± 32 (56)	Moderate
0.70 µM/plate	153 ± 10 (62)	Strong	243 ± 16 (67)	Strong
1.40 µM/plate	77 ± 12 (81)	Strong	161 ± 10 (78)	Strong
2.85 µM/plate	71 ± 9 (82)	Strong	106 ± 15 (86)	Strong
5.70 µM/plate	45 ± 18 (89)	Strong	47 ± 10 (94)	Strong
**Chrysin-ES**				
0.35 µM/plate	259 ± 16 (35)	Mild	431 ± 16 (42)	Moderate
0.70 µM/plate	221 ± 24 (45)	Moderate	221 ± 31 (70)	Strong
1.40 µM/plate	136 ± 8 (66)	Strong	111 ± 8 (85)	Strong
2.85 µM/plate	73 ± 5 (82)	Strong	53 ± 93 (93)	Strong
5.70 µM/plate	43 ± 19 (89)	Strong	25 ± 11 (97)	Strong

^a^ The data are indicated as mean ± SD of the number of revertant colonies of triplicate plates per treatment from two independent experiments. ^b^ Values in brackets indicate % mutagenic inhibition. ND: not determined.

**Table 8:** The anti-mutagenicity of chrysin and chrysin-ES on the urethane (URE)-induced mutations expressed as a reduction in mutant wing spots in *D. melanogaster* derived from trans-heterozygous *mwh*+/+*flr^3^* larvae of the ST cross.

Sample	Frequency of Mutant Spots per Individual (Number of Spots) ^a^				Inhibition (%)	Inhibitory Classification
	Small Single (1–2 Cells)	Large Single (>2 Cells)	Twin (m = 5)	Total (m = 2)		
**Negative control**	1.00 (40)	0 (0)	0 (0)	1.00 (40)	ND	ND
**URE**	3.70 (148) +	0.15 (6) +	0.05 (2) +	3.90 (156) +	ND	ND
**10%** **EtOH in PBS**	1.43 (57) i	0 (0) i	0 (0) i	1.43 (57) i	ND	ND
**Chrysin**						
20 μg/mL	0.23 (9)	0 (0)	0 (0)	0.23 (9)	94	Strong
40 μg/mL	0.33 (13)	0.03 (1)	0 (0)	0.35 (14)	91	Strong
80 μg/mL	0.35 (14)	0 (0)	0 (0)	0.35 (14)	91	Strong
120 μg/mL	0.55 (22)	0.03 (1)	0 (0)	0.56 (23)	86	Strong
**Chrysin-ES**						
20 μg/mL	0.18 (7)	0 (0)	0 (0)	0.18 (7)	95	Strong
40 μg/mL	0.68 (27)	0 (0)	0 (0)	0.68 (27)	83	Strong
80 μg/mL	0.30 (12)	0.03 (1)	0 (0)	0.33 (13)	92	Strong
120 μg/mL	0.55 (22)	0.03 (1)	0.03 (1)	0.60 (24)	85	Strong

^a^ Statistical diagnoses using estimation of spot frequencies and confidence limits according to Frei and Würgler (1988) [28] for comparison with deionized water (DI); “+” = positive; “i” = inconclusive. Probability levels: α = β = 0.05. One-sided statistical test. URE = urethane; EtOH = ethanol; PBS = phosphate buffer saline, pH 7.4; ND: not determined.

**Table 9:** The anti-mutagenicity of chrysin and chrysin-ES on the urethane (URE)-induced mutations, expressed as a reduction in mutant wing spots in *D. melanogaster* derived from trans-heterozygous *mwh*+/+*flr^3^* larvae in the HB cross.

Sample	Frequency of Mutant Spots per Individual (Number of Spots) ^a^				Inhibition (%)	Inhibitory Classification
	Small Single (1–2 Cells)	Large Single (>2 Cells)	Twin (m = 5)	Total (m = 2)		
**Negative control**	0.30 (12)	0.03 (1)	0.05 (2)	0.38 (15)	ND	ND
**URE**	8.20 (328) +	1.60 (64) +	0.70 (28) +	10.6 (424) +	ND	ND
**10%** **EtOH in PBS**	0.18 (7) −	0 (0) i	0 (0) i	0.18 (7) −	ND	ND
**Chrysin**						
20 μg/mL	1.55 (62)	0.15 (6)	0 (0)	1.70 (68)	84	Strong
40 μg/mL	1.75 (70)	0 (0)	0 (0)	1.75 (70)	84	Strong
80 μg/mL	0.88 (35)	0.03 (1)	0 (0)	0.90 (36)	92	Strong
120 μg/mL	1.08 (43)	0.08 (3)	0.03 (1)	1.18 (47)	89	Strong
**Chrysin-ES**						
20 μg/mL	1.95 (78)	0.10 (4)	0.10 (4)	2.15 (86)	80	Strong
40 μg/mL	1.83 (73)	0.25 (10)	0.10 (4)	2.18 (87)	79	Strong
80 μg/mL	0.70 (28)	0.03 (1)	0.03 (1)	0.75 (30)	93	Strong
120 μg/mL	0.58 (23)	0.05 (2)	0 (0)	0.63 (25)	94	Strong

^a^ Statistical diagnoses using estimation of spot frequencies and confidence limits, according to Frei and Würgler (1988) [28], for comparison with deionized water (DI); “+” = positive; “−” = negative; “i” = inconclusive. Probability levels: α = β = 0.05. One-sided statistical test. URE = urethane; EtOH = ethanol; PBS = phosphate buffer saline, pH 7.4; ND: not determined.

**Table 10:** The anti-mutagenicity of chrysin and chrysin-ES on the mitomycin C (MMC)-induced mutations, expressed as reduction in mutant wing spots in *D. melanogaster* derived from trans-heterozygous *mwh*+/+*flr^3^* larvae in the ST cross.

Sample	Frequency of Mutant Spots per Individual (Number of Spots) ^a^				Inhibition (%)	Inhibitory Classification
	Small Single (1–2 Cells)	Large Single (>2 Cells)	Twin (m = 5)	Total (m = 2)		
**Negative control**	0.93 (37)	0.08 (3)	0 (0)	1.00 (40)	ND	ND
**MMC**	6.93 (277) +	2.08 (83) +	0.75 (30) +	9.75 (390) +	ND	ND
**0.0125% DMSO**	0.08 (3) −	0.03 (1) −	0 (0) i	0.1 (4) −	ND	ND
**10%** **EtOH in PBS**	0.88 (35) −	0 (0) −	0 (0) i	0.88 (35) −	ND	ND
**Chrysin**						
20 μg/mL	8.05 (322)	1.45 (58)	0.35 (14)	9.85 (394)	−1	NA
40 μg/mL	4.98 (199)	1.35 (54)	0.53 (21)	6.85 (274)	30	Weak
80 μg/mL	4.48 (179)	1.63 (65)	0.73 (29)	6.83 (273)	30	Weak
120 μg/mL	1.50 (60)	0.58 (23)	0.18 (7)	2.25 (90)	77	Strong
**Chrysin-ES**						
20 μg/mL	4.25 (170)	1.50 (60)	0.18 (7)	5.93 (237)	39	week
40 μg/mL	2.85 (114)	1.75 (70)	0.25 (10)	4.85 (194)	50	moderate
80 μg/mL	4.00 (80)	0.40 (8)	0.08 (3)	4.55 (91)	53	moderate
120 μg/mL	1.40 (56)	0.18 (7)	0.05 (2)	1.63 (65)	83	Strong

^a^ Statistical diagnoses using estimation of spot frequencies and confidence limits according to Frei and Würgler (1988) [28] for comparison with deionized water (DI); “+” = positive; “−” = negative; “i” = inconclusive. Probability levels: α = β = 0.05. One-sided statistical test. URE = urethane; EtOH = ethanol; PBS = phosphate buffer saline, pH 7.4. ND: not determined, NA: not applicable.

**Table 11:** The anti-mutagenicity of chrysin and chrysin-ES on the mitomycin C (MMC)-induced mutations, expressed as reduction in mutant wing spots in *D. melanogaster* derived from trans-heterozygous *mwh*+/+*flr^3^* larvae in the HB cross.

Sample	Frequency of Mutant Spots per Individual (Number of Spots) ^a^				Inhibition (%)	Inhibitory Classification
	Small Single (1–2 Cells)	Large Single (>2 Cells)	Twin (m = 5)	Total (m = 2)		
**Negative control**	0.13 (5)	0.03 (1)	0 (0)	0.15 (6)	ND	ND
**MMC**	2.90 (116) +	1.43 (57) +	0.55 (22) +	4.88 (195) +	ND	ND
**0.0125% DMSO**	0.20 (8) i	0 (0) i	0 (0) i	0.20 (8) i	ND	ND
**10%** **EtOH in PBS**	0.15 (6) i	0 (0) i	0 (0) i	0.15 (6) i	ND	ND
**Chrysin**						
20 μg/mL	3.70 (148)	0.85 (34)	0.10 (4)	4.65 (186)	5	Negligible
40 μg/mL	1.63 (65)	0.18 (7)	0.13 (5)	1.93 (77)	60	Strong
80 μg/mL	0.98 (39)	0.08 (3)	0 (0)	1.05 (42)	79	Strong
120 μg/mL	0.55 (22)	0.03 (1)	0 (0)	0.58 (23)	88	Strong
**Chrysin-ES**						
20 μg/mL	0.35 (14)	0.03 (1)	0 (0)	0.38 (15)	92	Strong
40 μg/mL	0.18 (7)	0 (0)	0 (0)	0.18 (7)	96	Strong
80 μg/mL	0.13 (5)	0 (0)	0 (0)	0.13 (5)	97	Strong
120 μg/mL	0.28 (11)	0 (0)	0 (0)	0.28 (11)	94	Strong

^a^ Statistical diagnoses using estimation of spot frequencies and confidence limits according to Frei and Würgler (1988) [28] for comparison with deionized water (DI); “+” = positive; “i” = inconclusive. Probability levels: α = β = 0.05. One-sided statistical test. URE = urethane; EtOH = ethanol; PBS = phosphate buffer saline, pH 7.4. ND: not determined.

**Table 12:** The anti-mutagenicity of chrysin and chrysin-ES on ethyl methanesulfonate (EMS)-induced mutations, expressed as a reduction in mutant wing spots in *D. melanogaster* derived from trans-heterozygous *mwh*+/+*flr^3^* larvae in the ST cross.

Sample	Frequency of Mutant Spots per Individual (Number of Spots) ^a^				Inhibition (%)	Inhibitory Classification
	Small Single (1–2 Cells)	Large Single (>2 Cells)	Twin (m = 5)	Total (m = 2)		
**Negative control**	0.93 (37)	0.08 (3)	0 (0)	1.00 (40)	ND	ND
**EMS**	5.60 (224) +	1.75 (70) +	0.45 (18) +	7.80 (312) +	ND	ND
**10%** **EtOH in PBS**	0.90 (36) −	0 (0) −	0 (0) i	0.90 (36) −	ND	ND
**Chrysin**						
20 μg/mL	4.88 (195)	1.28 (51)	0.20 (8)	6.35 (254)	19	Negligible
40 μg/mL	4.28 (117)	0.93 (37)	0.35 (14)	4.20 (168)	46	Moderate
80 μg/mL	3.50 (140)	0.35 (14)	0.10 (4)	3.95 (158)	49	Moderate
120 μg/mL	3.40 (136)	0.88 (35)	0.28 (11)	4.55 (182)	42	Moderate
**Chrysin-ES**						
20 μg/mL	4.93 (197)	0.63 (25)	0.20 (8)	5.75 (230)	26	Weak
40 μg/mL	4.08 (163)	0.58 (23)	0.13 (5)	4.78 (191)	39	Weak
80 μg/mL	3.80 (152)	0.43 (17)	0.30 (12)	4.53 (181)	42	Moderate
120 μg/mL	2.73 (109)	0.55 (22)	0.20 (8)	3.48 (139)	55	Moderate

^a^ Statistical diagnosis using estimation of spot frequencies and confidence limits according to Frei and Würgler (1988) [28] for comparison with deionized water (DI); “+” = positive; “−” = negative; “i” = inconclusive. Probability levels: α = β = 0.05. One-sided statistical test. URE = urethane; EtOH = ethanol; PBS = phosphate buffer saline, pH 7.4. ND: not determined.

**Table 13:** The anti-mutagenicity of chrysin and chrysin-ES on ethyl methanesulfonate (EMS)-induced mutations, expressed as a reduction in mutant wing spots in *D. melanogaster* derived from trans-heterozygous *mwh*+/+*flr^3^* larvae in the HB cross.

Sample	Frequency of Mutant Spots per Individual (Number of Spots) ^a^				Inhibition (%)	Inhibitory Classification
	Small Single (1–2 Cells)	Large Single (>2 Cells)	Twin (m = 5)	Total (m = 2)		
**Negative control**	0.03 (1)	0.05 (2)	0 (0)	0.08 (3)	ND	ND
**EMS**	10.00 (400) +	0.40 (16) +	0 (0) i	10.40 (416)	ND	ND
**10%** **EtOH in PBS**	0.18 (7) +	0 (0) i	0.03 (1) i	0.20 (8) i	ND	ND
**Chrysin**						
20 μg/mL	5.3 (212)	1.63 (65)	0.88 (35)	7.80 (312)	25	Weak
40 μg/mL	6.03 (241)	0.78 (31)	0.33 (13)	7.13 (285)	31	Weak
80 μg/mL	3.23 (129)	0.73 (29)	0.23 (9)	4.18 (167)	60	Moderate
120 μg/mL	2.20 (88)	0.20 (8)	0.13 (5)	2.53 (101)	76	Strong
**Chrysin-ES**						
20 μg/mL	5.68 (227)	0.33 (13)	0.20 (8)	6.20 (248)	40	Weak
40 μg/mL	3.95 (158)	0.95 (38)	0.18 (7)	5.08 (203)	51	Moderate
80 μg/mL	2.68 (107)	0.15 (6)	0.08 (3)	2.90 (116)	72	Strong
120 μg/mL	2.10 (84)	0.23 (9)	0.20 (8)	2.53 (101)	76	Strong

^a^ Statistical diagnoses using estimation of spot frequencies and confidence limits according to Frei and Würgler (1988) [28] for comparison with deionized water (DI); “+” = positive; “i” = inconclusive. Probability levels: α = β = 0.05. One-sided statistical test. URE = urethane; EtOH = ethanol; PBS = phosphate buffer saline, pH 7.4. ND: not determined.

**Table 14:** The anti-mutagenicity of chrysin and chrysin-ES on the *N*-nitrosomethylurea (NMU)-induced mutations, expressed as a reduction in mutant wing spots in *D. melanogaster* derived from trans-heterozygous *mwh*+/+*flr^3^* larvae in the ST cross.

Sample	Frequency of Mutant Spots per Individual (Number of Spots) ^a^				Inhibition (%)	Inhibitory Classification
	Small Single (1–2 Cells)	Large Single (>2 Cells)	Twin (m = 5)	Total (m = 2)		
**Negative control**	0.18 (7)	0.05 (2)	0 (0)	0.23 (9)	ND	ND
**Nitrite**	0.05 (2) −	0 (0)	0.03 (1) i	0.08 (3) −	ND	ND
**Methylurea**	0.08 (3) −	0.03 (1) i	0 (0) i	0.10 (4) −	ND	ND
**NMU**	1.70 (68) +	0.10 (4) i	0.20 (8) +	2.00 (80) +	ND	ND
**10%** **EtOH in PBS**	1.08 (43) −	0 (0)	0 (0)	1.08 (43)	ND	ND
**Chrysin**						
20 μg/mL	0.15 (6)	0.03 (1)	0.05 (2)	0.23 (9)	89	Strong
40 μg/mL	0.18 (7)	0 (0)	0 (0)	0.18 (7)	91	Strong
80 μg/mL	0.18 (7)	0 (0)	0 (0)	0.18 (7)	91	Strong
120 μg/mL	0.13 (5)	0.08 (3)	0 (0)	0.20 (8)	90	Strong
**Chrysin-ES**						
20 μg/mL	0.10 (4)	0.05 (2)	0 (0)	0.15 (6)	93	Strong
40 μg/mL	0.08 (3)	0.03 (1)	0 (0)	0.10 (4)	95	Strong
80 μg/mL	0.15 (6)	0 (0)	0 (0)	0.15 (6)	93	Strong
120 μg/mL	0.28 (11)	0.03 (1)	0 (0)	0.30 (12)	85	Strong

^a^ Statistical diagnoses using estimation of spot frequencies and confidence limits according to Frei and Würgler (1988) [28] for comparison with deionized water (DI); “+” = positive; “−” = negative; “i” = inconclusive. Probability levels: α = β = 0.05. One-sided statistical test. URE = urethane; EtOH = ethanol; PBS = phosphate buffer saline, pH 7.4. ND: not determined.

**Table 15:** The anti-mutagenicity of chrysin and chrysin-ES on the *N*-nitrosomethylurea (NMU)-induced mutations expressed as a reduction in mutant wing spots in *D. melanogaster* derived from trans-heterozygous *mwh*+/+*flr^3^* larvae in the HB cross.

Sample	Frequency of Mutant Spots per Individual (Number of Spots) ^a^				Inhibition (%)	Inhibitory Classification
	Small Single (1–2 Cells)	Large Single (>2 Cells)	Twin (m = 5)	Total (m = 2)		
**Negative control**	0.73 (29)	0.08 (3)	0 (0)	0.80 (32)	ND	ND
**Nitrite**	0.25 (10) −	0.03 (1) i	0 (0) i	0.28 (11) i	ND	ND
**Methylurea**	0.18 (7) −	0.03 (1) i	0.03 (1) i	0.23 (9) i	ND	ND
**NMU**	1.43 (57) −	0.40 (16) +	0.10 (4) i	1.93 (77) +	ND	ND
**10%** **EtOH in PBS**	0.23 (9) i	0 (0) i	0 (0) i	0.23 (9) i	ND	ND
**Chrysin**						
20 μg/mL	0.78 (25)	0.09 (3)	0 (0)	0.88 (28)	54	Moderate
40 μg/mL	0.30 (12)	0.05 (2)	0.03 (1)	0.38 (15)	80	Strong
80 μg/mL	0.32 (9)	0.04 (1)	0 (0)	0.36 (10)	81	Strong
120 μg/mL	0.05 (2)	0.05 (2)	0 (0)	0.10 (4)	95	Strong
**Chrysin-ES**						
20 μg/mL	0.73 (29)	0.03 (1)	0 (0)	0.75 (30)	61	Strong
40 μg/mL	0.90 (36)	0.05 (2)	0.05 (2)	1.00 (40)	48	Moderate
80 μg/mL	0.38 (15)	0.10 (4)	0.03 (1)	0.50 (20)	74	Strong
120 μg/mL	0.25 (10)	0.05 (2)	0 (0)	0.30 (12)	84	Strong

^a^ Statistical diagnoses using estimation of spot frequencies and confidence limits according to Frei and Würgler (1988) [28] for comparison with deionized water (DI); “+” = positive; “−” = negative; “i” = inconclusive. Probability levels: α = β = 0.05. One-sided statistical test. URE = urethane; EtOH = ethanol; PBS = phosphate buffer saline, pH 7.4. ND: not determined.

## Data Availability

The datasets used and/or analyzed during the current study are available from the corresponding author upon reasonable request.

## Supplementary Materials

The following supporting information can be downloaded at: https://www.mdpi.com/article/10.3390/nano14121001/s1, Supplementary Figure S1: The conceptual framework of chrysin-ES formulation; Supplementary Table S1: Quantification of chrysin content and entrapment efficacy of chrysin-ES stored at 25 °C for five weeks; Supplementary Table S2: Clinical observation and health examination results (number of abnormal animals/all animals); Supplementary Table S3: Results of mortality and morbidity and gross finding. Reference [11] is cited in the Supplementary Materials.
