# Supplementary material for: Multi-Endpoint Toxicological Assessment of Chrysin Loaded Oil-in-Water Emulsion System in Different Biological Models
Source: Nanomaterials (Basel). 2024 Jun 8;14(12):1001. doi: 10.3390/nano14121001 (PMC11206519; doi:10.3390/nano14121001)
Supplement: Supplementary file 1 [file nanomaterials-14-01001-s001.zip › nanomaterials-3000838-supplementary.pdf]

Multi-Endpoint Toxicological Assessment of Chrysin Loaded Oil-in-Water Emulsion System in Different Biological Models

Pornsiri Pitchakarn [1,†], Pisamai Ting [2,†], Pensiri Buacheen [1], Jirarat Karinchai [1], Woorawee Inthachat [2], Boonrat Chantong [3], Uthaiwan Suttisansanee [2], Onanong Nuchuchua [4] and Piya Temviriyanukul [2,*]

[1] Department of Biochemistry, Faculty of Medicine, Chiang Mai University, Muang Chiang Mai, Chiang Mai 50200, Thailand; pornsiri.p@cmu.ac.th (P.P.); pensiri.bua@cmu.ac.th (P.B.); jirarat.ka@cmu.ac.th (J.K.)
[2] Institute of Nutrition, Mahidol University, Salaya, Nakhon Pathom 73170, Thailand; pisamai.ting@gmail.com (P.T.); woorawee.int@mahidol.ac.th (W.I.); uthaiwan.sut@mahidol.ac.th (U.S.)
[3] Department of Pre-Clinical and Applied Animal Science, Faculty of Veterinary Science, Mahidol University, Salaya, Phutthamonthon, Nakhon Pathom 73170, Thailand; boonrat.cha@mahidol.ac.th
[4] National Nanotechnology Center (NANOTEC), National Science and Technology Development Agency (NSTDA), Khlong Luang, Pathum Thani 12120, Thailand; onanong@nanotec.or.th
* Correspondence: piya.tem@mahidol.ac.th
† These authors contributed equally to this work.

Supplementary Figure S1. The conceptual framework of chrysin-ES. Chrysin is a flavonoid with great health benefits but has low bioaccessibility and bioavailability. Chrysin emulsion system may cope with that issue. Thus, the development and optimization of chrysin emulsion systems were performed, resulting in several formulations of chrysin emulsions. After intensive characterization, only chrysin-ES (code chrysin-NE1), was chosen for further studies, and a large batch production was prepared. The chrysin-ES from the large batch production was subjected to (i) characterization of chrysin-ES, (ii) bioaccessibility of chrysin-ES, (iii) safety assessment, and (iv) genotoxicity and anti-genotoxicity. The results obtained from (i) and (ii) were published in Foods (doi: 10.3390/foods10081912), while the results obtained from (iii) and (iv) are in this study.

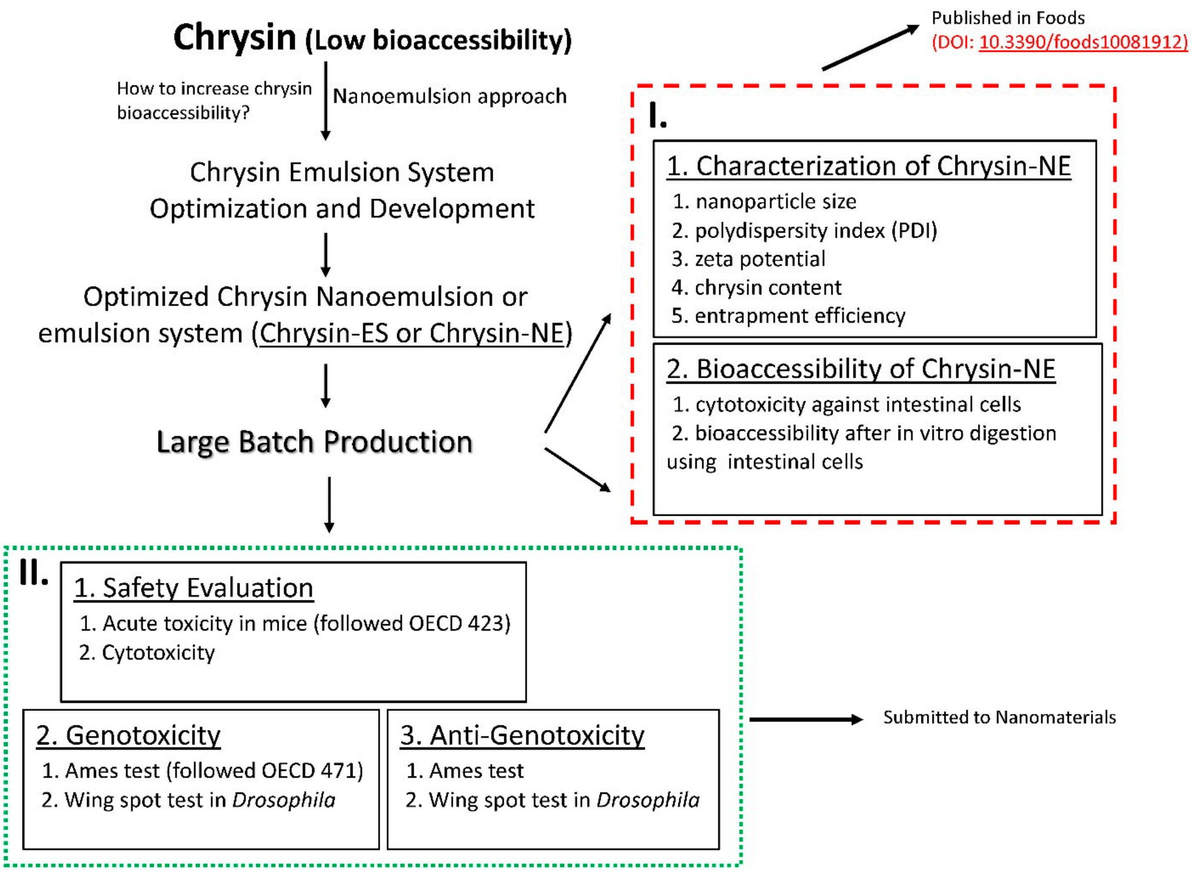

Supplementary Table S1. Quantification of chrysin content, entrapment efficacy and nanoparticle characterization of chrysin-ES at the production day and after stored at 25°C for five weeks (data from Ting et al., 2021).

Chrysin-ES	Chrysin content (µg/g)	Entrapment efficiency (% *w*/*w*)	Size (nm)	PDI	Zeta potential (mV)
Production day	174.21 ± 1.98	100.29 ± 0.53	161 ± 1.96	0.21 ± 0.01	–32 ± 0.061
5 weeks	179.54 ± 0.71	101.75 ± 2.76	173 ± 3.11	0.27 ± 0.01	–30 ± 2.06

Reference:

Ting, P.; W. Srinuanchai; U. Suttisansanee; S. Tuntipopipat; S. Charoenkiatkul; K. Praengam; B. Chantong; P. Temviriyanukul, O. Nuchuchua. Development of Chrysin Loaded Oil-in-Water Nanoemulsion for Improving Bioaccessibility. Foods 2021, 10. DOI: 10.3390/foods10081912.

Supplementary Table S2. Clinical observation and health examination results (Number of abnormal animals/all animals).

Time after administration	Chrysin-ES (mg/kg body weight)			
	300 (Group1)	300 (Group 2)	2000 (Group 3)	2000 (Group 4)
1 day	0/3	0/3	0/3	0/3
2 days	0/3	0/3	0/3	0/3
3 days	0/3	0/3	0/3	0/3
4 days	0/3	0/3	0/3	0/3
5 days	0/3	0/3	0/3	0/3
6 days	0/3	0/3	0/3	0/3
7 days	0/3	0/3	0/3	0/3
8 days	0/3	0/3	0/3	0/3
9 days	0/3	0/3	0/3	0/3
10 days	0/3	0/3	0/3	0/3
11 days	0/3	0/3	0/3	0/3
12 days	0/3	0/3	0/3	0/3
13 days	0/3	0/3	0/3	0/3
14 days	0/3	0/3	0/3	0/3

Supplementary Table S3. Results of mortality and morbidity and gross finding.

Chrysin-ES (mg/kg body weight)	Animal number	Mortality and Morbidity	Gross finding
300 (Group 1)	1	No	No remarkable lesions
	2	No	No remarkable lesions
	3	No	No remarkable lesions
300 (Group 2)	4	No	No remarkable lesions
	5	No	Uterus; clear fluid distension
	6	No	No remarkable lesions
2000 (Group 3)	7	No	No remarkable lesions
	8	No	No remarkable lesions
	9	No	No remarkable lesions
2000 (Group 4)	10	No	No remarkable lesions
	11	No	No remarkable lesions
	12	No	No remarkable lesions
